# A mixed-method service evaluation of health information exchange in England: technology acceptance and barriers and facilitators to adoption

**DOI:** 10.1186/s12913-021-06771-z

**Published:** 2021-07-25

**Authors:** Fiona Watkinson, Kanika I. Dharmayat, Nikolaos Mastellos

**Affiliations:** 1 Department of Primary Care and Public Health, School of Public Health, Imperial College London, London, UK; 2 Imperial Centre for Cardiovascular Disease Prevention (ICCP), Department of Primary Care and Public Health, School of Public Health, Imperial College London, London, UK

**Keywords:** Health information exchange, Technology acceptance, Technology adoption, Unified theory of acceptance and use of technology, Normalisation process theory

## Abstract

**Background:**

The need for information exchange and integrated care has stimulated the development of interoperability solutions that bring together patient data across the health and care system to enable effective information sharing. Health Information Exchange (HIE) solutions have been shown to be effective in supporting patient care, however, user adoption often varies among users and care settings. This service evaluation aimed to measure user acceptance of HIE and explore barriers and facilitators to its wider uptake.

**Methods:**

A mixed-method study design was used. A questionnaire was developed using the Unified Theory of Acceptance and Use of Technology and administered to HIE users to assess technology acceptance. Pearson Chi^2^ tests were used to examine differences in acceptance between user groups and care settings. Web-based, semi-structured interviews were conducted drawing on the Normalisation Process Theory to explore barriers and facilitators to adoption. Interview data were analysed thematically using the Framework Approach.

**Results:**

A total of 105 HIE users completed the survey and another 12 participated in the interviews. Significant differences were found in HIE acceptance between users groups and care settings, with high adopters demonstrating higher acceptance and social care users showing lower acceptance. Participants identified several drivers to adoption, including increased information accessibility, better care coordination, informed decision-making, improved patient care, reduced duplication of procedures, and time and cost savings. However, they also highlighted a number of barriers, such as lack of awareness about the solution and its value, suboptimal communication strategies, inadequate training and lack of resources for knowledge dissemination, absence of champions to support the implementation, lack of end-user involvement in the implementation and evaluation of HIE, unclear accountability and responsibility for the overall success of the programme, and patient confidentiality concerns.

**Conclusions:**

Working to better engage stakeholders, considering the needs of users from different care settings, providing users with training resources and support to increase their knowledge and confidence in using the system, developing implementation strategies to seek user feedback and monitor performance, and using communication strategies to increase awareness of the product and its value, can help improve uptake and adoption of HIE.

**Supplementary Information:**

The online version contains supplementary material available at 10.1186/s12913-021-06771-z.

## Background

Provision of health care across health systems involves the use of a variety of specialists and services. This necessitates the need for health and care information sharing and the availability of standards for secure data exchange across multiple Electronic Health Records (EHR) systems to develop new solutions that bring together health and social care information from siloed sources to support person-centred care. Health Information Exchange (HIE) involves electronic mobilisation of patient information within and across organisations and between various EHR systems according to locally and/or nationally recognised standards to enable informed decision-making and improve the quality of healthcare in a population [1]. HIE solutions have been shown to provide a variety of benefits to health care systems, such as minimising gaps in patient histories, avoiding unnecessary duplication of procedures and investigations, reducing hospital admissions, referrals, and costs, and improving immunisation rates, patient safety, experience and outcomes [2–6].

These improvements are particularly important considering the shift to more integrated care models that require multiple providers in a local area to work together to achieve greater integration of health and social care services and improve population health. HIE solutions have been shown to be particularly effective in connecting social care services, such as hospice care centres, which typically exist as siloed systems separate to the National Health Service (NHS). For example, the implementation of HIE in St. Josephs Hospice in Hackney, England, allowed the hospice to access over 100,000 records from 12 East London health and care organisations through the East London Patient Record (eLPR), while enabling eLPR to gain access to personalised urgent care plans designed through St. Josephs Hospice [7].

Despite the established benefits of HIE, user acceptance remains a prominent barrier in the uptake of HIE systems. Completeness and timeliness of information, patient privacy, workflow considerations, user awareness and usability are well established factors influencing adoption [8, 9]. Also, as the value of HIE solutions increases through the increased number of services and providers that are interconnected, peer influence and network effects are key to user acceptance and adoption [10, 11].

Various models have been described to aid assessment of user acceptance of technology, including the technology acceptance model (TAM). This model utilises four measures of behaviour variables (Perceived Ease of Use, Perceived Usefulness, Attitude Toward Use, and Behavioural Intention to Use) assessed through questionnaires to determine the usage and adoption of technology [8, 9]. An extension of TAM, TAM2, was developed to include social influence and cognitive processes (Subjective Norm, Voluntariness, Image, Experience, Job Relevance, Output Quality, Result Demonstrability) within the TAM model [10], and more recently, TAM3 was developed to account for additional factors influencing human decision making, such as Computer Self-efficacy, Computer Anxiety, Computer Playfulness, and Perceptions of External Control [11]. Whilst TAM is considered to be a good predictor model to determine usage and acceptability, it has been deemed to not be a good predictor of actual use of technology by individuals [12]. To consolidate these limitations, a derivation of TAM known as the unified theory of acceptance and use of technology (UTAUT) was developed. Using four constructs (Performance Expectancy, Effort Expectancy, Social Influence, and Facilitating Conditions) which are moderated by gender, age, experience, and voluntariness of use, UTAUT attempts to explain real-world intention to use an IT system [13].

Alongside understanding user acceptance, establishing the perceived barriers and facilitators to uptake of health technology is imperative given these systems are known to possess a variety of barriers and facilitators to their wider uptake despite general acceptance and adoption within a certain community. Whilst various models have been proposed to aid understanding barriers and facilitators [14], the normalisation process theory (NPT) assesses multiple sociological constructs (Coherence, Cognitive Preparation, Collective Action, and Reflexive Monitoring) in order to determine factors that either inhibit or promote the incorporation of a new complex healthcare intervention into routine use, and has been shown to be a robust and reliable measure to use for studies focused on localised implementation [15–17].

While small-scale studies of HIE acceptance have shown positive results, currently, no studies have assessed user acceptance across a variety of contexts within England to account for the diverse needs that exist within different health and care settings and regions. This study assesses user acceptance of Cerner’s HIE solution across England and explores barriers and facilitators to its wider implementation. Capturing the perspectives of HIE users is hugely powerful as it demonstrates its value to their peers and patients and identifies barricades to adoption as well as opportunities for improvement.

## Methods

### Study aim

This study was designed to measure user acceptance of Cerner’s HIE solution across 15 NHS Trusts in England and explore barriers and facilitators to its wider uptake. At the time of the study (June–July 2020), adoption varied amongst users and care settings in the participating sites. It was therefore deemed important to generate insights into the observed differences in HIE system uptake by analysing the relationship between user acceptance, contextual factors, and adoption.

### Study design

A mixed-method study design was adopted to address the study aims, consisting of development and administration of an end-user survey to quantify HIE system acceptance and web-based interviews to generate qualitative data surrounding perceived barriers and facilitators to uptake.

### Participants and setting

**Fig. 1:**
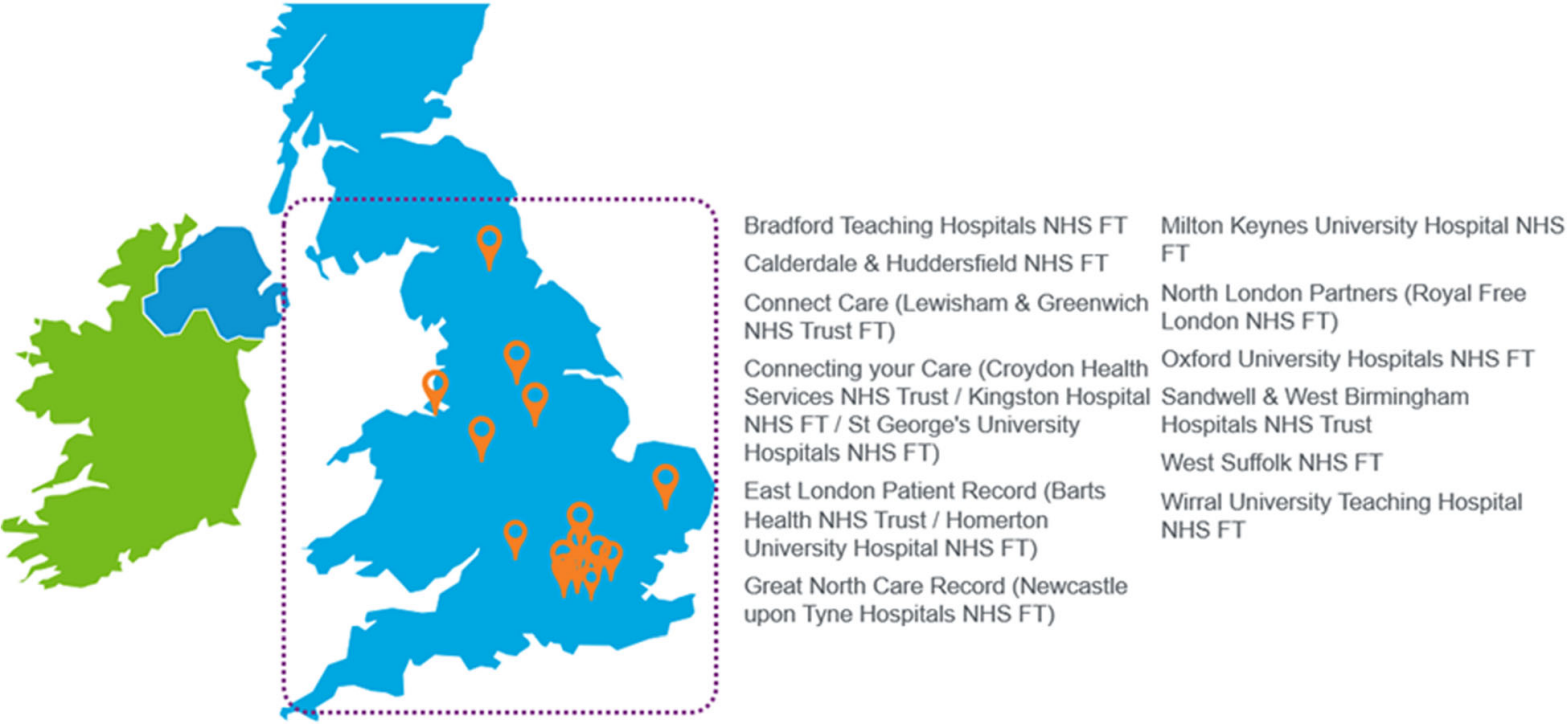
NHS Trusts in England utilising Cerner’s HIE platform

Participants were users and implementation teams of Cerner’s HIE platform from 15 NHS Trusts in England. Cerner’s HIE system is a cloud-based, vendor-agnostic solution enabling information sharing across acute, primary, community, social care, and mental health settings. Users can access the HIE solution as a ‘click-through’ from their EHR system and get real-time access to patient data from disperse sources. When this study was conducted, the HIE system had been implemented across 15 NHS Trusts in England covering a population of ≈13 million patients (Fig. 1).

### Data collection

A questionnaire was designed to collect demographic data, such as age, gender and occupation, and assess users’ perceptions of HIE through a five-point Likert scale (ranging from ‘strongly disagree’ to ‘strongly agree’) designed using the UTAUT framework (supplementary file 1) [13]. The survey included two TAM constructs (i.e. Job Relevance and Perceived Enjoyment) to assess whether HIE was relevant to the daily tasks of users from diverse care settings and whether end-users enjoyed using the system. Following questionnaire design, a link to the online survey, which was developed using Imperial College London’s Qualtrics® survey tool, was sent to the participating NHS organisations who were responsible for administering the survey to their staff to protect participants’ anonymity. The survey was designed to take less than 10 min to complete. The questionnaire remained open for 4 weeks, from mid-June 2020 to mid-July 2020, with reminder emails to complete the survey sent weekly following initial questionnaire distribution.

An opt-in within the questionnaire allowed participants to volunteer for a follow-up virtual interview exploring barriers and facilitators to the use of the HIE system by providing their email address. The research team reached out via email to provide participants with a sign-up link for an interview time. All participants were provided with a participant information sheet to brief them on stipulations of the study. Interview questions and format were designed using NPT constructs (supplementary file 2) [17]. While NPT provided a framework for the interview structure, general questions on overall perception of barriers and facilitators to the implementation of HIE system within health systems were also introduced. Interviews were semi-structured and conducted using Microsoft Teams virtual conferencing software and lasted between 15 and 30 min. Interviews were recorded with permission of the interviewee to ensure each interview could be accurately transcribed later for analysis. Recordings were deleted following transcription. The transcriptions are stored securely in servers at Imperial College London.

### Data analysis

Survey data were exported from the Imperial College London Qualtrics® system, cleaned, coded and analysed using Stata® Version 15. Due to the small sample size, all five-point Likert scales were condensed to three levels (i.e. ‘Disagree’, consisting of ‘Strongly Disagree’ and ‘Disagree’; ‘Neither Agree nor Disagree’; and ‘Agree’, consisting of ‘Agree’ and ‘Strongly Agree’) to achieve a clearer delineate the direction of responses amongst user groups (i.e. low, medium and high level users). Data were analysed for all respondents and by user group: high-level (using HIE 5+ times per week), mid-level (3–4 times per week) and low-level (1–2 times per week) users. Those groups were defined by Cerner Corporation following analysis of monthly HIE system usage data from the participating sites. Descriptive statistics were used to describe the sample characteristics and overall HIE system acceptance. Pearson’s Chi^2^ tests with a significance level of *p* ≤ .05 were used to assess differences between user groups by demographic characteristics and technology acceptance.

Interview data were transcribed verbatim and analysed thematically using the Framework Approach [18]. This involved familiarisation with the data through reading the interview transcripts, identification of a priori and emerging themes using the notes from the transcripts, indexing of relevant portions of interview data to corresponding thematic categories, charting, mapping and interpretation by summarising priori and emergent themes using the charted themes and relevant quotations from the transcripts.

## Results

### Demographics

A total of 115 HIE users completed the survey. Of those, 10 were excluded as they only completed the demographic portion of the survey. The demographics of the 105 respondents are shown in Table 1. The majority were high-level users, followed by mid-level and low-level users. There were no significant differences between user groups across all demographic variables.

**Table 1:** Survey demographics

	Total		Low-level user		Medium-level user		High-level user		*P*
	n	(%)	n	(%)	n	(%)	n	(%)	
	105	(100)	14	(13.3)	16	(15.2)	75	(71.4)	
Gender									.219
Male	34	(32.4)	8	(57.1)	7	(43.8)	43	(57.3)	
Female	70	(66.7)	6	(42.9)	8	(50)	31	(41.3)	
Prefer Not to Say	1	(1.0)	0	(0)	1	(6.2)	1	(1.3)	
Age									.616
18–44 years	58	(55.2)	4	(28.6)	5	(31.3)	25	(33.3)	
45–64 years	45	(42.9)	10	(71.4)	10	(62.5)	50	(66.7)	
65 and older	2	(1.9)	0	(0)	1	(6.2)	0	(0)	
Care setting									.943
Primary Care	24	(22.9)	3	(21.4)	5	(31.3)	16	(21.3)	
Hospital	44	(41.9)	4	(28.6)	6	(37.5)	34	(45.3)	
Social Care Services	8	(7.6)	2	(14.3)	1	(6.3)	5	(6.7)	
Mental Health Facility	9	(8.6)	2	(14.3)	1	(6.3)	6	(8.0)	
Third Sector	2	(1.9)	0	(0)	0	(0)	2	(2.7)	
Community Care Services	18	(17.1)	3	(21.4)	3	(18.3)	12	(16.0)	
Occupation									.274
Doctor	37	(35.2)	6	(42.9)	4	(25.0)	27	(36.0)	
Nurse	20	(19.1)	1	(7.1)	2	(12.5)	17	(22.7)	
Midwife	1	(1.0)	0	(0)	1	(6.3)	0	(0)	
Change Manager	2	(1.9)	1	(7.1)	0	(0)	1	(1.3)	
Pharmacist	13	(12.4)	1	(7.1)	3	(18.7)	9	(12.0)	
Allied Health Professional	19	(18.1)	2	(14.3)	6	(37.5)	11	(14.7)	
Administrative/Clerical	1	(1.0)	0	(0)	0	(0)	1	(1.3)	
Practice Manager	4	(3.8)	1	(7.1)	0	(0)	3	(4.0)	
Other	8	(7.6)	2	(14.3)	0	(0)	6	(8.0)	

Twelve individuals participated in an interview. Most were females (*n* = 9) and represented the 45–65 year-old category (*n* = 7), with the rest being between 18 and 44 years old. Three participants were members of the implementation team, while the rest were end-users. Most interviewees (*n* = 7) were from an acute setting with representation from four more settings: mental health (*n* = 2), primary care (*n* = 1), community care (n = 1) and social care (*n* = 1). Most participants were doctors (*n* = 4), followed by allied health professionals (*n* = 3), nurses (*n* = 2), a pharmacist, a social worker, and a change manager.

### HIE system acceptance

Overall, technology acceptance (Table 2) was high with most users finding HIE useful (PE), easy to use (EE), relevant to their role (REL) and thus using it routinely (HT). In addition, participants showed a clear intention to use the HIE system regularly in the future, with some users also being keen to increase its use (BI). Enjoyment responses were mixed with 45% of users finding the HIE solution enjoyable to use and 40% providing a neutral response. However, respondents felt that there was no push from the top or other advocates of the HIE solution to use the system (SI). Also, the training provided was inadequate and there was no support available when users needed help with using the system (FC).

**Table 2:** HIE acceptance

Construct	Survey Question	Disagree	Neutral	Agree
		n (%)	n (%)	n (%)
**PE1**	HIE has saved me time at work	4 (3.8)	11 (10.5)	90 (85.7)
**PE2**	HIE has made my job easier	2 (1.9)	7 (6.7)	96 (91.4)
**PE3**	Using HIE helps me to be a better healthcare provider	2 (1.9)	14 (13.3)	89 (84.8)
**PE4**	Using HIE supports critical aspects of my patients’ healthcare	3 (2.9)	6 (5.7)	96 (91.4)
**PE5**	Using HIE enhances my effectiveness as a healthcare provider	2 (1.9)	12 (11.4)	91 (86.7)
**PE6**	Overall, HIE is useful to me in managing my patients’ healthcare	1 (1.0)	8 (7.6)	96 (91.4)
**EE1**	Learning how to use HIE was easy for me	5 (4.8)	11 (10.5)	89 (84.7)
**EE2**	It was easy for me to become skilful at using HIE	9 (8.6)	10 (9.5)	86 (81.9)
**EE3**	I find HIE easy to use	9 (8.6)	12 (11.4)	84 (80.0)
**PE1**	HIE is enjoyable to use	16 (15.2)	42 (40.0)	47 (44.8)
**REL1**	In my job, using HIE is important	1 (1.0)	1 (1.0)	103 (98.0)
**REL2**	The use of HIE is pertinent to many of my job-related tasks	2 (1.9)	10 (9.5)	93 (88.6)
**HT1**	The use of HIE has become a habit for me	6 (5.7)	13 (12.4)	86 (81.9)
**BI1**	I intend to use HIE regularly	0 (0)	4 (3.8)	101 (96.2)
**BI2**	I intend to increase the amount I use HIE in the future	0 (0)	33 (31.4)	72 (68.6)
**SI1**	People in my workplace promote the use of HIE	13 (12.4)	24 (22.9)	68 (64.7)
**SI2**	People who influence my behaviour at work use HIE	8 (7.6)	31 (29.5)	66 (62.9)
**SI3**	Most individuals in my workplace use HIE	9 (8.6)	31 (29.5)	65 (61.9)
**FC1**	I received adequate training when I began using HIE	53 (50.5)	22 (20.9)	30 (28.6)
**FC2**	I have the resources necessary to use HIE	7 (6.7)	16 (15.2)	82 (78.1)
**FC3**	I have the knowledge necessary to use HIE	4 (3.8)	15 (14.3)	86 (81.9)
**FC4**	HIE is compatible with the other electronic health systems I use at work	16 (15.2)	25 (23.8)	64 (61.0)
**FC5**	I can receive help when I have difficulties using HIE	39 (37.1)	37 (35.2)	29 (27.7)

*PE* Performance Expectancy, *EE* Effort Expectancy; *PE* Perceived Enjoyment; *REL* Job Relevance, *HT* Habit, *BI* Behavioural Intention, *SI* Social Influence, *FC* Facilitating Conditions.

Table 3 shows the breakdown of responses by user group. High adopters provided consistently more positive responses compared to low- and medium-level users. Significant differences were observed between user groups in terms of how relevant the HIE solution was in their job (REL2), whether using the system had become a habit for them (HT1), the impact of individuals who influence their behaviour on adoption (SI1, SI2), and the possession of the necessary knowledge to use the system (FC3).When collapsing the low- and medium-user groups into one category and comparing with the high adopters category, additional significant differences were revealed (Table 4). High adopters were more likely to believe that using the HIE system enhances their effectiveness in their job (PE5) and intended to use it regularly in the future (BI1).

**Table 3:** HIE acceptance between user groups

	Low-level users			Medium-level users			High-level users				
Construct	Disagree	Neutral	Agree	Disagree	Neutral	Agree	Disagree	Neutral	Agree	x^**2**^	***P***
	n (%)	n (%)	n (%)	n (%)	n (%)	n (%)	n (%)	n (%)	n (%)	(4, 105)	
PE1	2 (14.3)	1 (7.1)	11 (78.6)	1 (6.3)	2 (12.5)	13 (81.3)	1 (1.3)	8 (10.7)	66 (88)	5.87	.209
PE2	1 (7.1)	2 (14.3)	11 (78.6)	0 (0)	2 (12.5)	14 (87.5)	1 (1.3)	3 (4)	71 (94.7)	5.65	.227
PE3	0 (0)	3 (21.4)	11 (78.6)	1 (6.3)	4 (25)	11 (68.7)	1 (1.3)	7 (9.3)	67 (89.3)	5.93	.204
PE4	0 (0)	1 (7.1)	13 (92.9)	1 (6.3)	2 (12.5)	13 (81.2)	2 (2.7)	3 (4)	70 (93.3)	2.99	.559
PE5	0 (0)	2 (14.3)	12 (85.7)	0 (0)	5 (31.3)	11 (68.7)	2 (2.7)	5 (11.7)	68 (90.6)	8.62	.071
PE6	0 (0)	1 (7.1)	13 (92.)	0 (0)	3 (13.8)	13 (81.2)	1 (1.3)	4 (5.3)	70 (91.4)	3.73	.443
EE1	1 (7.1)	1 (7.1)	12 (85.7)	1 (6.3)	4 (25)	11 (68.7)	3 (4)	6 (8)	66 (88)	4.72	.317
EE2	1 (7.1)	0 (0)	13 (92.9)	3 (18.8)	4 (25)	9 (56.2)	5 (6.7)	6 (8)	64 (85.3)	9.42	.051
EE3	1 (7.1)	1 (7.1)	12 (85.7)	1 (6.3)	5 (31.2)	10 (62.5)	7 (9.3)	6 (8)	62 (82.7)	7.43	.115
PE1	2 (14.3)	7 (50)	5 (35.7)	2 (12.5)	8 (50)	6 (37.5)	12 (16)	27 (36)	36 (48)	1.79	.775
REL1	1 (7.1)	0 (0)	13 (92.9)	0 (0)	0 (0)	16 (100)	0 (0)	1 (1.3)	74 (98.7)	6.95	.139
REL2	1 (7.1)	2 (14.3)	11 (78.6)	0 (0)	5 (31.3)	11 (68.7)	1 (1.3)	3 (4)	71 (94.7)	14.3	.006
HT1	3 (21.4)	3 (21.4)	8 (57.1)	3 (18.8)	3 (18.8)	10 (62.5)	0 (0)	7 (9.3)	68 (90.7)	19.59	.001
BI1	0 (0)	1 (7.1)	13 (92.9)	0 (0)	2 (12.5)	14 (87.5)	0 (0)	1 (1.3)	74 (98.7)	4.98	.083
BI2	0 (0)	6 (42.9)	8 (57.1)	0 (0)	6 (37.5)	10 (62.5)	0 (0)	21 (28)	54 (72)	1.53	.465
SI1	5 (35.7)	4 (28.6)	5 (35.7)	1 (6.3)	3 (18.8)	12 (75)	7 (9.3)	17 (22.7)	51 (68)	9.73	.045
SI2	3 (21.4)	6 (42.9)	5 (35.7)	1 (6.3)	7 (43.8)	8 (50)	4 (5.3)	18 (24)	53 (70.7)	9.56	.048
SI3	0 (0)	6 (42.9)	8 (57.1)	2 (12.5)	3 (18.8)	11 (68.8)	7 (9.3)	22 (29.3)	46 (61.3)	3.19	.527
FC1	8 (57.1)	3 (21.4)	3 (21.4)	7 (43.8)	4 (25)	5 (31.3)	38 (50.7)	15 (20)	22 (29.3)	0.73	.947
FC2	1 (7.1)	2 (14.3)	11 (78.6)	1 (6.3)	3 (18.7)	12 (75)	5 (6.7)	11 (14.7)	59 (78.6)	0.19	.996
FC3	1 (7.1)	4 (28.57)	9 (64.5)	1 (6.3)	5 (31.3)	10 (62.5)	2 (2.7)	6 (8)	67 (89.3)	9.98	.041
FC4	1 (7.1)	4 (28.6)	9 (64.3)	4 (25)	3 (18.8)	9 (56.2)	11 (14.7)	18 (24)	46 (61.3)	2.01	.734
FC5	4 (28.6)	6 (42.9)	4 (28.6)	3 (18.6)	8 (50)	5 (31.3)	32 (42.7)	23 (35.7)	20 (26.7)	4.12	.390

**Table 4:** HIE acceptance between user groups

	Low/medium-level users			High-level users				
Construct	Disagree	Neutral	Agree	Disagree	Neutral	Agree	x^**2**^	***P***
	n (%)	n (%)	n (%)	n (%)	n (%)	n (%)	(4, 105)	
**PE5**	0 (0)	7 (23.3)	23 (76.7)	2 (2.7)	5 (6.7)	68 (90.7)	6.49	.039
**REL2**	0 (0)	5 (31.3)	11 (68.7)	1 (1.3)	3 (4.0)	71 (94.7)	14.3	.006
**HT1**	3 (18.8)	3 (18.8)	10 (62.5)	0 (0)	7 (9.3)	68 (90.7)	19.59	.001
**BI1**	0 (0)	3 (10)	27 (90)	0 (0)	1 (1.3)	74 (98.7)	4.4	.036
**SI2**	4 (13.3)	13 (43.3)	13 (43.3)	4 (5.3)	18 (24)	53 (70.7)	7.06	.029
**FC3**	1 (6.3)	5 (31.3)	10 (62.5)	2 (2.7)	6 (8)	67 (89.3)	9.98	.041

Significant differences were also found between care settings (Table 5). The Performance Expectancy category (PE4, PE5, PE6) showed notable differences, with only 62.5% of social care respondents agreeing that HIE was helpful for managing patient care as compared to 91.7% in primary care, 100% in hospitals, and 88.9% in mental health and community care. Similar rates were seen in enhancing effectiveness as a provider and supporting critical aspects of healthcare, with only 37.5 and 75% of social services agreeing with these statements, respectively. A significant difference was also noted in the Social Influence category (SI3), with only 12.5% of social care and 50% of community care users indicating that the HIE solution was used widely in their workplace. Finally, respondents had significantly different opinions with regards to two Facilitating Conditions (FC2, FC5), with half of social care respondents indicating that they had the resources to use the system, and all third sector users saying that they could receive support when they had difficulties using the HIE solution.

**Table 5:** HIE acceptance by care setting

	Primary Care (*n* = 24)			Acute Care (*n* = 44)			Community Care (*n* = 18)			Mental Health (n = 9)			Social Care (*n* = 8))			Third Sector (n = 2)				
Question	Disagree	Neutral	Agree	Disagree	Neutral	Agree	Disagree	Neutral	Agree	Disagree	Neutral	Agree	Disagree	Neutral	Agree	Disagree	Neutral	Agree	x^2^	*P*
	n (%)	n (%)	n (%)	n (%)	n (%)	n (%)	n (%)	n (%)	n (%)	n (%)	n (%)	n (%)	n (%)	n (%)	n (%)	n (%)	n (%)	n (%)	(10, 105)	
PE1	1 (4.2)	4 (16.7)	19 (79.2)	2 (4.5)	4 (9.1)	38 (86.4)	0 (0)	2 (11.1)	16 (88.9)	1 (11.1)	1 (11.1)	7 (77.8)	0 (0)	0 (0)	8 (100.0)	0 (0)	0 (0)	2 (100.0)	4.86	.900
PE2	0 (0)	3 (12.5)	21 (87.5)	1 (2.3)	1 (2.3)	42 (95.4)	0 (0)	2 (11.1)	16 (88.9)	1 (11.1)	1 (11.1)	7 (77.8)	0 (0)	0 (0)	8 (100.0)	0 (0)	0 (0)	2 (100.0)	9.39	.496
PE3	1 (4.2)	3 (12.5)	20 (83.3)	0 (0)	3 (6.8)	41 (93.2)	0 (0)	3 (16.7)	15 (83.3)	0 (0)	1 (11.1)	8 (88.9)	1 (12.5)	3 (37.5)	4 (50.0)	0 (0)	1 (50.0)	1 (50.0)	15.69	.109
PE4	0 (0)	2 (8.3)	22 (91.7)	0 (0)	0 (0)	44 (100.0)	2 (11.1)	1 (5.6)	15 (83.3)	1 (11.1)	0 (0)	8 (88.9)	0 (0)	2 (25.0)	6 (75.0)	0 (0)	1 (50.0)	1 (50.0)	25.16	.005
PE5	0 (0)	3 (12.5)	21 (87.5)	0 (0)	2 (4.5)	42 (95.5)	0 (0)	2 (11.1)	16 (88.9)	1 (11.1)	0 (0)	8 (88.9)	1 (12.5)	4 (50.0)	3 (37.5)	0 (0)	1 (50.0)	1 (50.0)	29.24	.001
PE6	0 (0)	2 (8.3)	22 (91.7)	0 (0)	0 (0)	44 (100.0)	0 (0)	2 (11.1)	16 (88.9)	1 (11.1)	0 (0)	8 (88.9)	0 (0)	3 (37.5)	5 (62.5)	0 (0)	1 (50.0)	1 (50.0)	30.58	.001
EE1	2 (8.3)	4 (16.7)	18 (75.0)	1 (2.3)	5 (11.4)	38 (86.4)	1 (5.6)	1 (5.6)	16 (88.9)	0 (0)	1 (11.1)	8 (88.9)	1 (12.5)	0 (0)	7 (87.5)	0 (0)	0 (0)	2 (100.0)	5.55	.852
EE2	4 (16.7)	4 (16.7)	16 (66.7)	2 (4.6)	3 (6.8)	39 (88.6)	2 (11.1)	1 (5,6)	15 (83.3)	0 (0)	2 (22.2)	7 (77.8)	1 (12.5)	0 (0)	7 (87.5)	0 (0)	0 (0)	2 (100.0)	9.34	.500
EE3	3 (12.5)	5 (20.8)	16 (66.7)	3 (6.8)	4 (9.1)	37 (84.1)	0 (0)	2 (11.1)	16 (88.9)	1 (11.1)	1 (11.1)	7 (77.8)	2 (25.0)	0 (0)	6 (75.0)	0 (0)	0 (0)	2 (100.0)	9.04	.529
PE1	6 (25)	9 (37.5)	9 (37.5)	4 (9.1)	13 (29.5)	27 (61.4)	3 (16.7)	8 (44.4)	7 (38.9)	1 (11.1)	7 (77.8)	1 (11.1)	2 (25.0)	4 (50.0)	2 (25.0)	0 (0)	1 (50.0)	1 (50.0)	14.43	.154
REL1	0 (0)	0 (0)	24 (100.0)	0 (0)	0 (0)	44 (100.0)	1 (5.6)	0 (0)	17 (94.4)	0 (0)	1 (11.1)	8 (88.9)	0 (0)	0 (0)	8 (100.0)	0 (0)	0 (0)	2 (100.0)	15.63	.111
REL2	0 (0)	6 (25.0)	18 (75.0)	0 (0)	2 (4.6)	42 (95.4)	1 (5.6)	2 (11.1)	15 (83.3)	1 (11.1)	0 (0)	8 (88.9)	0 (0)	0 (0)	8 (100.0)	0 (0)	0 (0)	2 (100.0)	16.73	.081
HT1	1 (4.2)	4 (16.7)	19 (79.2)	2 (4.6)	4 (9.1)	38 (86.4)	2 (11.1)	4 (22.2)	12 (66.7)	0 (0)	0 (0)	9 (100.0)	1 (12.5)	1 (12.5)	6 (75.0)	0 (0)	0 (0)	2 (100.0)	7.03	.722
BI1	0 (0)	1 (4.2)	23 (95.8)	0 (0)	2 (4.6(	42 (95.4)	0 (0)	1 (5.6)	17 (94.4)	0 (0)	0 (0)	9 (100.0)	0 (0)	0 (0)	8 (100)	0 (0)	0 (0)	2 (100.0)	0.976	.965
BI2	0 (0)	5 (20.8)	19 (79.2)	0 (0)	12 (27.3)	32 (72.7)	0 (0)	8 (44.4)	10 (55.6)	0 (0)	4 (44.4)	5 (55.6)	0 (0)	4 (50.0)	4 (50.0)	0 (0)	0 (0)	2 (100.0)	5.92	.314
SI1	3 (12.5)	3 (12.5)	18 (75.0)	5 (11.4)	9 (20.4)	30 (68.2)	2 (11.1)	8 (44.4)	8 (44.4)	2 (22.2)	1 (11.1)	6 (66.7)	1 (12.5)	3 (37.5)	4 (50.0)	0 (0)	0 (0)	2 (100.0)	9.94	.445
SI2	3 (12.5)	6 (25)	15 (62.5)	1 (2.3)	12 (27.3)	31 (70.4)	1 (5.6)	8 (44.4)	9 (50.0)	1 (11.1)	3 (33.3)	5 (55.6)	2 (25.0)	2 (25.0)	4 (50.0)	0 (0)	0 (0)	2 (100.0)	9.86	.453
SI3	3 (12.5)	5 (20.8)	16 (66.7)	1 (2.3)	11 (25.0)	32 (72.7)	1 (5.6)	8 (44.4)	9 (50.0)	3 (33.3)	1 (11.1)	5 (55.6)	1 (12.5)	6 (75.0)	1 (12.5)	0 (0)	0 (0)	2 (100.0)	23.93	.008
FC1	13 (54.2)	6 (25.0)	5 (20.8)	20 (45.5)	8 (18.2)	16 (36.4)	11 (61.1)	5 (27.8)	2 (11.1)	6 (66.7)	1 (11.1)	2 (22.2)	3 (37.5)	2 (25.0)	3 (37.5)	0 (0)	0 (0)	2 (100.0)	11.36	.330
FC2	2 (8.3)	6 (25.0)	16 (66.7)	0 (0)	3 (6.8)	41 (93.2)	2 (11.1)	3 (16.7)	13 (72.2)	0 (0)	2 (22.2)	7 (77.8)	2 (25.0)	2 (25.0)	4 (50.0)	1 (50.0)	0 (0)	1 (50.0)	21.27	.019
FC3	2 (8.3)	7 (29.2)	15 (62.5)	1 (2.3)	3 (6.8)	40 (90.9)	0 (0)	3 (16.7)	15 (83.3)	0 (0)	1 (11.1)	8 (88.9)	1 (12.5)	1 (12.5)	6 (75.0)	0 (0)	0 (0)	2 (100.0)	11.85	.295
FC4	7 (29.2)	7 (29.2)	10 (41.6)	3 (6.8)	9 (20.5)	32 (72.7)	3 (16.7)	4 (22.2)	11 (61.1)	1 (11.1)	3 (33.3)	5 (55.6)	1 (12.5)	2 (25.0)	5 (62.5)	1 (50.0)	0 (0)	1 (50.0)	10.74	.378
FC5	12 (50.0)	9 (37.5)	3 (12.5)	11 (25.0)	15 (34.1)	18 (40.9)	7 (38.9)	9 (50.0)	2 (11.1)	4 (44.4)	4 (44.4)	1 (11.1)	5 (62.5)	0 (0)	3 (37.5)	0 (0)	0 (0)	2 (100.0)	21.53	.018

### Barriers and facilitators to adoption

Expectations varied across participants, with many not being aware of the HIE solution at the time when HIE was implemented and therefore having no expectations. Stakeholder involvement during the implementation was relatively low with most participants stating that clinical teams and end-users were not consulted about the product prior to its implementation and that they were only made aware through an email notifying them of the upcoming implementation. Following deployment, end-users said that they had not been contacted for feedback and indicated that they would like to provide feedback through some organised forum.

“*I’m a senior social worker so I’ve got my own case load and supervise people with their own case-loads, and none of us at our level within my team were involved in that.*"

All participants agreed that end-users did not receive any training beyond email communications in a few cases. However, some also said that training on the system was not necessary. Participants suggested using super-users or champions to disseminate knowledge of the product, as well as reference materials such as demo videos and user manuals that use terminology which is appropriate for different regions and care settings. Others suggested regular reminders from Information Technology (IT) teams to help with learning the system and utilising it appropriately. Notably, most participants felt that a course or active training class would not be necessary for existing users, but training on induction would be helpful.

One of the nurses said:

“*It would be probably quite nice to be able to refer to something and say can I find this, could I use it for this, could I use it for that?*”

Another participant (doctor) mentioned:

*“I don’t think sending me on a course would help me, I just need to practice it a bit more and be reminded."*

End-users felt that use of the HIE solution is down to each individual practitioner with no accountability for uptake. However, implementation team participants stated that IT and change team members held their own teams accountable for diffusion and dispersed demo sessions and sent reminders to users to increase adoption. This was also acknowledged by some end-users.

A nurse participant said:

*“Funnily enough, our IT teams reminded us a few times for those who hadn’t used it so that was really good, that was quite clever."*

All participants indicated that the HIE system was useful in their work as it allows real-time information accessibility within seconds, saving them time, enabling better decision-making, improving patient safety, increasing efficiency, and reducing duplication of procedures.

One participant (pharmacist) stated:

*“It’s provided us a platform for innovative practice, better patients outcome, joining up systems which is so important, integrating different systems instead of working in isolation. Its saving us time so it translates into clinical efficiency as well.”*

However, some participants also felt that there is room for improvement, especially with increasing the area of HIE coverage and making the solution more user-friendly, particularly for social care and mental health users.

As a social worker mentioned:

*“Once you actually open the tab and go into HIE it doesn’t look like anything we use in adult social care. "*

Some participants recognised consent and privacy issues around patient confidentiality as a potential barrier. Others highlighted the importance of stakeholder engagement and improved communication strategies to increase awareness of the product and its value and thereby uptake. Communication was acknowledged as a key issue, with social care and mental health participants most consistently indicating a lack of awareness and suggesting better e-mail communications.

A doctor said:

*“What pains me is there not strategy for making sure that everybody knows what this is and how they can use it.”*

*“Nobody tells you about it, nobody values it particularly, it’s not high on anybody’s agenda.”*

Another participant (pharmacist) mentioned:

*“ I suppose communication. Email, or having a Microsoft meeting, inviting end-user, multidisciplinary team members to show what is HIE about, informing teams what HIE means, what sort of information we can access.”*

The main drivers and barriers to adoption are listed below.

**Table:** 

**Drivers to Adoption**	
• Increased information accessibility	
• Holistic view of patient care and better care coordination	
• Informed decision-making	
• Improved patient care/safety	
• Reduced duplication of procedures	
• Time and cost savings	
**Barriers to Adoption**	
• Lack of awareness about the solution and its value	
• Suboptimal communication strategies, especially in non-hospital settings	
• Inadequate training and lack of resources for knowledge dissemination	
• Absence of champions to support the implementation and uptake of the solution	
• Lack of end-user involvement in the implementation and evaluation of the solution	
• Unclear accountability and responsibility for the overall success of the programme	
• Privacy issues concerning patient confidentiality	

## Discussion

The primary objective of the study was to measure user acceptance of the HIE solution and analyse it by use level and setting through the administration of a survey utilising the UTAUT framework. The UTAUT has been frequently used across a variety of technologies and has shown to explain variance in use behaviour and intentions among end-users [19, 20]. A secondary objective was to explore barriers and facilitators to HIE system uptake using the NPT, which has been shown to be a valid and reliable method to assess implementation processes in a variety of contexts [16].

The findings show that most users perceived the HIE solution useful, easy to use and relevant to their role. Most respondents showed a clear intention to use it in the future, with some also being keen to increase its use. However, the survey also revealed some gaps in the implementation strategy which may have impeded adoption. There were also significant differences in user acceptance between user groups, indicating an association between technology acceptance and use of technology. Supplementary file 3 presents the survey and interview findings side-by-side.

Habitual use showed the largest statistically significant difference amongst user groups. Habit as a UTAUT construct has shown to be largely influenced by other factors such as importance to job, satisfaction, and experience with IT [21]. Given this, the difference in relevance between user groups that was also identified may be feeding into the lack of habit development observed in lower-lever user groups. Habitual usage amongst end-users is important to establish as it has shown to increase the uptake and use of digital technology [22].

Interviewees also indicated that HIE use was not embedded into their practice and expressed the need for further awareness, reminders about the product, and tools for its use to improve adoption. HIE organisations should develop communication strategies to ensure that all end-users are aware of the product, see the value in using it, and have the resources they need to optimise its use in specific contexts. The UK government may also consider incentivising HIE organisations as previous research has shown that, in addition to increasing awareness, financial incentives can positively influence HIE adoption and uptake [23].

Social Influence was also significantly different across user groups, with lower-level HIE users experiencing significantly lower peer influence than high-level users. Previous research on peer influence and network effects has shown that physicians are more likely to adopt HIE services when other physicians with whom they interact with use HIE [24, 25]. Social Influence, or subjective norm, relates to social proof theory, the idea of individuals looking to others within their environment for confirmation of behaviour, which has been well-established and applied to a variety of contexts, including digital technology and healthcare [26, 27]. The establishment of social proof is widely used as a marketing tool for companies and organisations, and should similarly be applied within HIE implementation strategy plans to increase acceptance and uptake of the system [28]. The use of a network of HIE ambassadors could promote subjective normalisation of the HIE solution and further increase user acceptance and adoption. There was also agreement across all user groups that training was inadequate. Similarly, all interviewees indicated that they received little to no training and that, while classroom-style teaching was not necessary, in situ training videos or resource manuals on how to use the product would be beneficial. The need for appropriate digital literacy was also highlighted, with high-level users indicating they have the knowledge necessary to use the HIE system significantly more than lower-level users. It is therefore important to provide users from different health facilities with training and support to increase their knowledge and confidence in using HIE and improve their attitudes towards using the system [29, 30].

Social care services exhibited lower levels of perceived usefulness and social influence. Social and community care services also indicated issues with training and support. Training to use the HIE solution was inadequate, and social care also noted they lacked the resources to properly use the system. The variations seen in user acceptance across care settings may be reflective of a lack of consideration of the profound differences between traditional and non-traditional care settings. Social and community care settings historically existed within silos separate from other NHS services; however, the demand for holistic care models has increased their integration with traditional NHS services [31]. As the system was primarily designed to be used in acute and primary care settings, considerations for services provided outside those settings were not made when designing the system. In fact, participants within social and community care services indicated issues with the user interface and with embedding the solution within their practices and noted that HIE did not look familiar to other systems used in those contexts. Therefore, it is important to tailor the design of the HIE solution to those settings and provide users with resources and tools to learn how to use the system effectively.

In line with findings from previous studies [3], use of the HIE system was found to result in time and cost savings, increased informed decision making, and collaborative care design. Several barriers were identified, including poor communication strategies and user awareness, inadequate training and resource provision, lack of stakeholder engagement, and privacy concerns. In developing implementation strategies, the consideration of all stakeholders must be addressed. Participants indicated that end-users were not involved pre- or post-implementation, and that system introduction was handled by IT and change teams. Engaging end-users early in the process but also post-implementation could result in higher levels of system awareness, approval, and utilisation [32]. As implementation of the HIE system expands, implementation strategies should be developed with collaborative end-users in order to minimise several barriers identified within this study and increase uptake and adoption.

Although the survey was distributed to a diverse range of HIE users, gender was not equally represented in the survey sample, which can be due to the fact that 77% of the NHS workforce is female [33]. In addition, participation to the survey was low due to the extreme pressures the NHS workforce was experiencing at the time of the study as a result of the COVID-19 pandemic. Also, most respondents were high adopters, which may have introduced some response bias due to the small representation of lower-level users in the study. Moreover, while the UTAUT framework was helpful for the design and understanding of the survey portion of the study, it may have oversimplified the relationships of the different constructs that feed into uptake and use of digital technology. Finally, the results of this study are not meant to be generalisable as service evaluations are primarily designed to assess current care standards and assist decision-making in particular settings, although they can be useful to others who are considering implementing a HIE solution.

Future studies should attempt to gain a larger sample size and generate findings that are more generalisable. Further investigation into the findings of this study surrounding inadequate training methods and limited support mechanisms for users would also be beneficial as this has shown to have a large impact on adoption and uptake of technologies [34]. The development and testing of new implementation plans for HIE to identify the most appropriate methodology for application on a national scale should also be addressed. An analysis of the policies that currently exist regarding how data is shared on HIE may also be beneficial to understanding privacy concerns that may be a barrier to widespread use.

## Conclusions

To the authors’ knowledge, this is the first service evaluation of HIE system acceptance across multiple NHS Trusts in England. The benefits of using the HIE solution were clear to end-users, however, there were also gaps in the implementation strategy, especially in non-traditional care settings. Working to better engage different stakeholders, considering the needs of end-users from social and community care settings, creating adequate training plans and support users to increase their knowledge and confidence in using HIE, developing implementation strategies to seek user feedback and monitor performance, and using communication strategies to increase awareness of the product and its value, could help overcome the barriers identified in this study and achieve maximum benefits as HIE expands to novel settings.

## Supplementary Information

**Additional file 1.** Survey Questions.

**Additional file 2.** Interview Questions.

**Additional file 3.** Summary of Findings.

## Data Availability

The datasets used and/or analysed during the current study are available from the corresponding author on reasonable request and with permission from the participating organisations.

## Abbreviations

BI: Behavioural Intention
EE: Effort Expectancy
EHR: Electronic Health Records
FC: Facilitating Conditions
GDPR: General Data Protection Regulation
HIE: Health Information Exchange
HT: Habit
IT: Information Technology
NHS: National Health Service
NPT: Normalisation Process Theory
PE: Performance Expectancy
REL: Job Relevance
SI: Social Influence
TAM: Technology Acceptance Model
UTAUT: Unified Theory of Acceptance and Use of Technology
