# Supplementary material for: A mixed-method service evaluation of health information exchange in England: technology acceptance and barriers and facilitators to adoption
Source: BMC Health Serv Res. 2021 Jul 25;21:737. doi: 10.1186/s12913-021-06771-z (PMC8310462; doi:10.1186/s12913-021-06771-z)
Supplement: Supplementary file 1 — Additional file 1. Survey Questions. [file 12913_2021_6771_MOESM1_ESM.docx]

| **Supplementary File 1: Survey Questions** | |
| --- | --- |
| **UTAUT Construct** | **Survey Items** |
| Performance Expectancy | HIE has saved me time at work |
|  | HIE has made my job easier |
|  | Using HIE helps me to be a better healthcare provider |
|  | Using HIE supports critical aspects of my patients' healthcare |
|  | Using HIE enhances my effectiveness as a healthcare provider |
|  | Overall, HIE is useful to me in managing my patients’ healthcare |
| Effort Expectancy | Learning how to use HIE was easy for me |
|  | It was easy for me to become skilful at using HIE |
|  | I find HIE easy to use |
| Perceived Enjoyment (ENJ)* | HIE is enjoyable to use |
| Job Relevance (REL)* | In my job, using HIE is important |
|  | The use of HIE is pertinent to many of my job-related tasks |
| Habit (HT) | The use of HIE has become a habit for me |
| Behavioural Intention (BI) | I intend to use HIE regularly |
|  | I intend to increase the amount I use HIE in the future |
| Social Influence | People in my workplace promote the use of HIE |
|  | People who influence my behaviour at work use HIE |
|  | Most individuals in my workplace use HIE |
| Facilitating Conditions (FC) | I received adequate training when I began using HIE |
|  | I have the resources necessary to use HIE |
|  | I have the knowledge necessary to use HIE |
|  | HIE is compatible with the other electronic health systems I use at work |
|  | I can receive help when I have difficulties using HIE |
|  | Viewing my patient's prescribed medications |
|  | Viewing my patient's timeline of encounters |

*TAM construct
