# Supplementary material for: A mixed-method service evaluation of health information exchange in England: technology acceptance and barriers and facilitators to adoption
Source: BMC Health Serv Res. 2021 Jul 25;21:737. doi: 10.1186/s12913-021-06771-z (PMC8310462; doi:10.1186/s12913-021-06771-z)
Supplement: Supplementary file 2 — Additional file 2. Interview Questions. [file 12913_2021_6771_MOESM2_ESM.docx]

| **Supplementary File 2: Interview Questions** | | |
| --- | --- | --- |
| **NPT Construct** | **Construct Element** | **Interview Guide Items** |
| Coherence | Differentiation | How is HIE different to previous practices for health information exchange? |
|  | Differentiation | Did you plan to make any changes in workflow to adjust for the use of HIE? |
|  | Communal Specification | What aims do you perceive the HIE system attempts to accomplish? |
|  | Communal Specification | What were the initial expectations for HIE? Has it achieved those expectations? |
|  | Individual Specification | Are you clear about your role in utilising HIE within your organisation? |
|  | Internalisation | What is the value/benefits of using HIE? |
| Cognitive Preparation | Initiation | Were all key stakeholders consulted before the implementation of HIE? Who was involved in the decision-making process? |
|  | Initiation | Which teams were involved in the implementation of HIE? Any teams that were not involved although they should have? |
|  | Enrolment | Were job responsibilities and roles influenced or changed by the use of HIE? |
|  | Legitimation | Do you feel end users were well considered during the implementation of HIE? |
|  | Activation | Have end users been involved in decisions about HIE post its implementation? |
| Collective Action | Interactional Workability | Was your training in using HIE adequate? Did you feel confident to use HIE after training? |
|  | Interactional Workability | Is there a set of best practices for using HIE in your workplace? Do these work well with your day-to-day work? |
|  | Relational Integration | Who has the overall accountability for the adoption and uptake of HIE within your organisation? |
|  | Skill Set Workability | Do you feel all appropriate individuals are using HIE to optimise the benefits? |
|  | Contextual Integration | Is the team well-resourced to achieve the goals set by your organisation? |
| Reflexive Monitoring | Systematisation | From your experience, where has HIE been helpful in your practice? Where it has been inhibitory and why? |
|  | Communal Appraisal | Can you think of ways that the implementation and uptake of HIE can be improved to enable more health and care professionals to use the platform? |
|  | Individual Appraisal | Has HIE improved your efficiency and the efficiency within your team or ward? |
|  | Reconfiguration | Are end users following the prescribed practices for the use of HIE? Have those changed over time? |
| **Other** | **Theme** | **Interview Guide Items** |
| General | Drivers to Uptake | What are the main drivers for the uptake of HIE within your organisation? |
| General | Barriers to Uptake | What are the main barriers to HIE’s wider uptake? |
