# Supplementary material for: A mixed-method service evaluation of health information exchange in England: technology acceptance and barriers and facilitators to adoption
Source: BMC Health Serv Res. 2021 Jul 25;21:737. doi: 10.1186/s12913-021-06771-z (PMC8310462; doi:10.1186/s12913-021-06771-z)
Supplement: Supplementary file 3 — Additional file 3. Summary of Findings. [file 12913_2021_6771_MOESM3_ESM.docx]

| **Supplementary File 3: Summary of Findings** | | | | |
| --- | --- | --- | --- | --- |
| **Theme** | **Sub-theme** | **Construct (Framework)** | **Qualitative** | **Quantitative** |
| Differences from previous practices | Efficient way of accessing health and care records | Coherence (NPT) | Previously, the majority of groups used phone calls and faxes to get information, which was wasting time. HIE allows for a space with single sign-on that integrates all care records increasing efficiency. |  |
| Changes in workflows and job roles | No role changes for health and care professionals | Coherence (NPT) | In healthcare roles, job roles were not significantly changed. |  |
|  | New roles in IT and change management teams | Coherence (NPT) | IT and change teams have adjusted significantly, incorporating HIE implementation teams and workflows. |  |
|  | Changes to patient care workflows | Coherence (NPT) | Health and care staff now look at patient details in HIE before approaching a new patient in A&E. |  |
|  | Seamless workflow integration | Coherence (NPT) | HIE integrated seamlessly into existing workflows and systems, making jobs easier in some cases. |  |
| Perceived aims, expectations and benefits | Shared aims | Coherence (NPT) | Aims of the product included easy access to patient information and improved continuity of care across systems. |  |
|  | Mixed expectations | Coherence (NPT) | Expectations of the product ranged from low, to none, to high. Largely HIE exceeded expectations. |  |
|  | Improved patient care | Coherence (NPT) | HIE has increased care effectiveness and efficiency. |  |
|  | Improved patient safety | Coherence (NPT) | HIE has improved patient safety. |  |
|  | Informed decision making | Coherence (NPT) | HIE has enabled clinicians to make more informed decisions. |  |
|  | Increased information accessibility | Coherence (NPT) | HIE has provided direct access to information from different systems. |  |
|  | Better care coordination | Coherence (NPT) | HIE has enabled better coordination through providing a holistic view of patient care. |  |
|  | Time and cost savings | Coherence (NPT) | HIE has reduced duplication of services. |  |
|  |  | Performance Expectancy (UTAUT) |  | 85.7% said that HIE has saved them time. |
| Stakeholder involvement in implementation | Lack of end-user involvement in implementation | Cognitive Preparation (NPT) | End-users were largely uninvolved in the implementation. This was conducted by Chief Clinical Informatics Officers, IT and change teams. Some clinicians were consulted. |  |
|  | No consultation with social care teams | Cognitive Preparation (NPT) | Social care involvement was particularly lacking. |  |
|  | Minimal communication about the product | Cognitive Preparation (NPT) | The system just appeared one day and users were not given a briefing on what to expect of the product. Vague email communications was all what was provided. |  |
|  | Lack of clinical risk team involvement | Cognitive Preparation (NPT) | Clinical risk team were not involved. |  |
| Stakeholder involvement post-implementation | Lack of end-user involvement in HIE evaluation | Cognitive Preparation (NPT) | The majority of end-users were not contacted for feedback. |  |
|  | Suboptimal communication strategies | Cognitive Preparation (NPT) | Communication needs improvement. Lately, clinical engagement groups were created to engage with end-users. |  |
|  | Low peer influence | Social Influence (UTAUT) |  | Only 62.9% said that peers who influence their behaviour use HIE. |
|  | Limited support from the top | Social Influence (UTAUT) |  | Only 64.7% felt that people within their organisation promote the use of HIE. |
| Training, best practices, uptake and accountability | Inadequate training and lack of resources for knowledge dissemination | Collective Action (NPT) | Most end-users did not receive training. Tools such as demo videos, guides, and super-users would be useful, especially for social care and mental health users. |  |
|  |  | Facilitating Conditions (UTAUT) |  | Only 28.6% said that training was adequate. |
|  | Intuitive system | Collective Action (NPT) | Some users felt that the system is intuitive and formal training is not needed. |  |
|  |  | Facilitating Conditions (UTAUT) |  | Although the majority of end-users did not receive training, 8 in 10 felt that they have the necessary knowledge and resources to use the system. |
|  |  | Effort Expectancy (UTAUT) |  | 84.7% said that learning how to use HIE was easy, and 81.9% felt that it was easy to become skilful at using HIE. |
|  | Lack of technical support | Collective Action (NPT) | The vendor should train on-site members to handle problems to streamline services. |  |
|  |  | Facilitating Conditions (UTAUT) |  | Only 27.7% said that they can receive support when required. |
|  | High user uptake | Collective Action (NPT) | HIE is widely used, there is high uptake through word of mouth. |  |
|  |  | Habit (UTAUT) |  | 8 in 10 respondents use HIE routinely. |
|  |  | Behavioural Intention (UTAUT) |  | 96.2% plan to use HIE regularly in the future, and 68.6% intend to increase its use. |
|  | Variations in uptake between users | Performance Expectancy (UTAUT) |  | There were significant differences in HIE acceptance between high and lower-level users. |
|  |  | Job Relevance (TAM) |  |  |
|  |  | Habit (UTAUT) |  |  |
|  |  | Behavioural Intention (UTAUT) |  |  |
|  |  | Social Influence (UTAUT) |  |  |
|  |  | Facilitating Conditions (UTAUT) |  |  |
|  | Variations in uptake across care settings | Collective Action (NPT) | Uptake varied, with hospitals widely using the product while social care and mental health not having embedded HIE use in practice. |  |
|  |  | Performance Expectancy (UTAUT) |  | Significant differences in HIE acceptance were found across settings, with social care respondents showing lower acceptance. |
|  |  | Social Influence (UTAUT) |  |  |
|  |  | Facilitating Conditions (UTAUT) |  |  |
|  | Unclear accountability and responsibility for the overall success of the programme | Collective Action (NPT) | There is no accountability for uptake within specific teams. IT change team sent reminders to use the system and held users accountable. |  |
| Experiences, efficiency, and engagement | HIE is useful | Reflexive Monitoring (NPT) | Most end-users find HIE helpful and useful in their practice. |  |
|  |  | Performance Expectancy (UTAUT) |  | Most participants found HIE useful (91.4%). |
|  | HIE is easy to use | Effort Expectancy (UTAUT) |  | Most (81%) thought that HIE is easy to use (81%). |
|  | HIE is efficient and effective | Reflexive Monitoring (NPT) | HIE has improved efficiency through reduced admin work and increased effectiveness. |  |
|  |  | Performance Expectancy (UTAUT) |  | Most said that HIE has made their job easier (91.4%), and supports critical aspects of their patients’ care (91.4%). They also felt that it has made them a better health and care provider (84.8%) and has enhanced their effectiveness (86.7%). |
|  | HIE is important | Job Relevance (TAM) |  | Respondents said that HIE is very important (98.0%) and pertinent (88.6%) to their job. |
|  | User interface needs improvement | Reflexive Monitoring (NPT) | System upgrades are needed to adjust glitches and improve the user interface, particularly for those in social and mental health care. |  |
|  |  | Perceived Enjoyment (TAM) |  | Only 44.8% thought that HIE is enjoyable to use. |
|  | Privacy issues concerning patient confidentiality | Reflexive Monitoring (NPT) | Some users may have patient confidentiality concerns. |  |
